# Emerging Antibiotic Resistance Patterns in a Neonatal Intensive Care Unit in Pune, India: A 2-Year Retrospective Study

**DOI:** 10.3389/fped.2022.864115

**Published:** 2022-06-10

**Authors:** Mubashir Hassan Shah, Samuel McAleese, Sandeep Kadam, Tushar Parikh, Umesh Vaidya, Sonali Sanghavi, Julia Johnson

**Affiliations:** ^1^Department of Pediatrics, Government Medical College, Srinagar, India; ^2^Division of Neonatology, Department of Pediatrics, Johns Hopkins University School of Medicine, Baltimore, MD, United States; ^3^Division of Neonatology, Department of Pediatrics, King Edward Memorial Hospital & Research Centre, Pune, India; ^4^Department of Microbiology, King Edward Memorial Hospital & Research Centre, Pune, India; ^5^Department of International Health, Johns Hopkins Bloomberg School of Public Health, Baltimore, MD, United States

**Keywords:** neonatal sepsis, bloodstream infection, meningitis, antimicrobial resistance, colistin resistance

## Abstract

**Objective:**

Treating neonatal bloodstream infections and meningitis in South Asia remains difficult given high rates of antimicrobial resistance (AMR). To evaluate changing epidemiology of neonatal infections, we assessed pathogen-specific and clinical features of culture-proven infections in neonates admitted to a neonatal intensive care unit (NICU) in Pune, India.

**Materials and Methods:**

This retrospective cohort study was performed in the King Edward Memorial Hospital and Research Center NICU over 2 years between January 1, 2017 and December 31, 2018. We included all neonates admitted to the NICU with positive blood or cerebrospinal fluid cultures. Demographic, clinical, and microbiologic data were collected from the medical record. We reviewed antimicrobial susceptibility testing (AST) of all isolates.

**Results:**

There were 93 culture-positive infections in 83 neonates, including 11 cases of meningitis. Fifteen (18%) neonates died. Gram-negative pathogens predominated (85%) and AST showed 74% resistance to aminoglycosides, 95% resistance to third/fourth generation cephalosporins, and 56% resistance to carbapenems. Resistance to colistin was present in 30% of *Klebsiella pneumoniae* isolates. Birth weight <1,000 g [odds ratio (OR) 6.0, *p* < 0.002], invasive respiratory support (OR 7.7, *p* = 0.001), and antibiotics at the time of culture (OR 4.2, *p* = 0.019) were associated with increased risk of mortality. Rates of AMR to all major antibiotic classes were similar between early onset and late onset infections. There was no association between carbapenem resistance and mortality.

**Conclusion:**

In our NICU in India, there are high rates of AMR among Gram-negative pathogens that are predominantly responsible for infections. Given higher colistin resistance in this cohort than previously reported, hospitals should consider routinely testing for colistin resistance.

## Introduction

Neonatal sepsis causes a large proportion of all neonatal deaths worldwide (1). India reports higher rates of neonatal infections than other low- and middle-income countries (LMIC), and a recent review of neonatal infections in India reported a case fatality rate of 50% for culture-positive sepsis (2, 3). Compounding the difficulty and complexity of treating neonatal sepsis is antimicrobial resistance (AMR); as antibiotic use has increased and broader spectrum antibiotics are used more frequently, AMR has risen (4). Previously published longitudinal data from our neonatal intensive care unit (NICU) in western India from 2006 to 2008 already showed a worrying increase in multidrug-resistant (MDR) infection over the 2-year study period as well as persistently high rates of cephalosporin and carbapenem resistance (5). A more recent systematic review from 2019 on AMR in neonatal sepsis in South Asia (including 69 studies from India) shows that these challenges are ongoing and more widespread (6). This review reported rates of resistance between 67 and 86% to first-line drugs recommended by the World Health Organization (WHO), such as ampicillin, gentamicin, and third-generation cephalosporins, as well as high (50–70%) degree of MDR in isolates throughout India and South Asia.

AMR surveillance studies guide institutional infection prevention practices, help national efforts to standardize recommendations, and inform future implementation research. These studies can also inform practices outside the regions and countries being studied. Human travel and globalization have contributed to the global spread of select mutations that confer colistin resistance, reinforcing the notion that AMR is not only a local or regional concern (7). Given the worsening trends in AMR, ongoing surveillance is vital to better assess risk factors for infection and the appropriateness of commonly used empiric antibiotic regimens. The objective of this study was to evaluate pathogen-specific and clinical features of culture-proven infections in neonates admitted to the NICU at a tertiary care healthcare facility in western India.

## Materials and Methods

### Patient Selection and Clinical Data

This retrospective cohort study was conducted at King Edward Memorial Hospital & Research Center (KEMHRC), a 550-bed multispecialty tertiary care teaching hospital in urban Pune, India, with a 50-bed Level III NICU. As part of the largest non-governmental hospital in Pune and a major referral center for the state of Maharashtra, the Department of Obstetrics and Gynecology at KEMHRC performs over 3,000 deliveries annually including high-risk deliveries. Approximately 1,000 infants—predominantly born at KEMHRC—are admitted to the NICU annually, although the NICU also serves as a referral center for smaller facilities throughout Maharashtra. This two-year study included all neonates admitted to the NICU between January 1, 2017, and December 31, 2018 with a documented positive culture from blood or cerebrospinal fluid (CSF). Culture specimens were collected and processed according to routine laboratory standards in KEMHRC's nationally accredited microbiology laboratory. Data were manually extracted from the hospital's electronic medical record using a standardized case report form. Demographic, birth, and clinical data were collected, including gestational age, birth weight, presence of central line at time of positive culture, relevant clinical diagnoses, and disposition. Microbiologic data were collected, including organism isolated, results of antibiotic susceptibility testing (AST), and time to culture positivity.

### Diagnostic and Microbiological Criteria

We examined all culture-proven infections in neonates. Culture-proven infections were defined as positive blood or CSF culture. Blood and CSF samples of minimum 1 ml were collected using aseptic technique; samples were immediately transported to the in-hospital laboratory for automated processing using the BD FX™ system (BD, Franklin Lakes, NJ, USA). Cultures that tested positive were subcultured on blood agar, MacConkey agar, and chocolate agar (CSF cultures only). Positive cultures with the same organism from both blood and CSF were considered as part of the same culture-positive infection. Multiple positive cultures from the same neonate were considered part of the same culture-positive infection unless a distinct organism was isolated, or the culture was drawn more than 7 days after the prior culture. Early onset blood stream infection (BSI) was defined as positive blood culture in the first 72 h of life, and early onset meningitis was defined as positive CSF culture in the first 72 h of life. Late onset BSI and meningitis were defined as positive culture from blood or CSF, respectively, after 72 h of life. Necrotizing enterocolitis (NEC) was defined as stage II or greater on modified Bell's criteria (8). Retinopathy of prematurity (ROP) was defined as any stage per International Committee for Classification of Retinopathy of Prematurity (ICROP) criteria (9). Intraventricular hemorrhage was defined according to the Papile grading classification (10). AST for all antibiotics was performed on the Vitek 2® system (BioMérieux, Inc, Hazelwood, MO, USA). Susceptibility testing results were interpreted according to minimum inhibitory concentration breakpoints set by the Clinical and Laboratory Standards Institute (CLSI, Malvern, PA, USA). Antibiotic resistance was defined as resistance or intermediate susceptibility on AST; resistance to a class of antibiotics was defined as resistance or intermediate susceptibility to any antimicrobial in that class.

### Statistical Analysis

Descriptive analysis was performed on the study population. Statistical analysis was performed with Stata 15.1 (StataCorp, College Station, TX, USA). Univariate regression using a Pearson χ^2^-test was used to assess the associations between clinical risk factors, carbapenem resistance, and mortality.

### Ethics Statement

This study was approved by the KEMHRC Ethics Committee (ID No. 2108).

## Results

**Figure 1:**
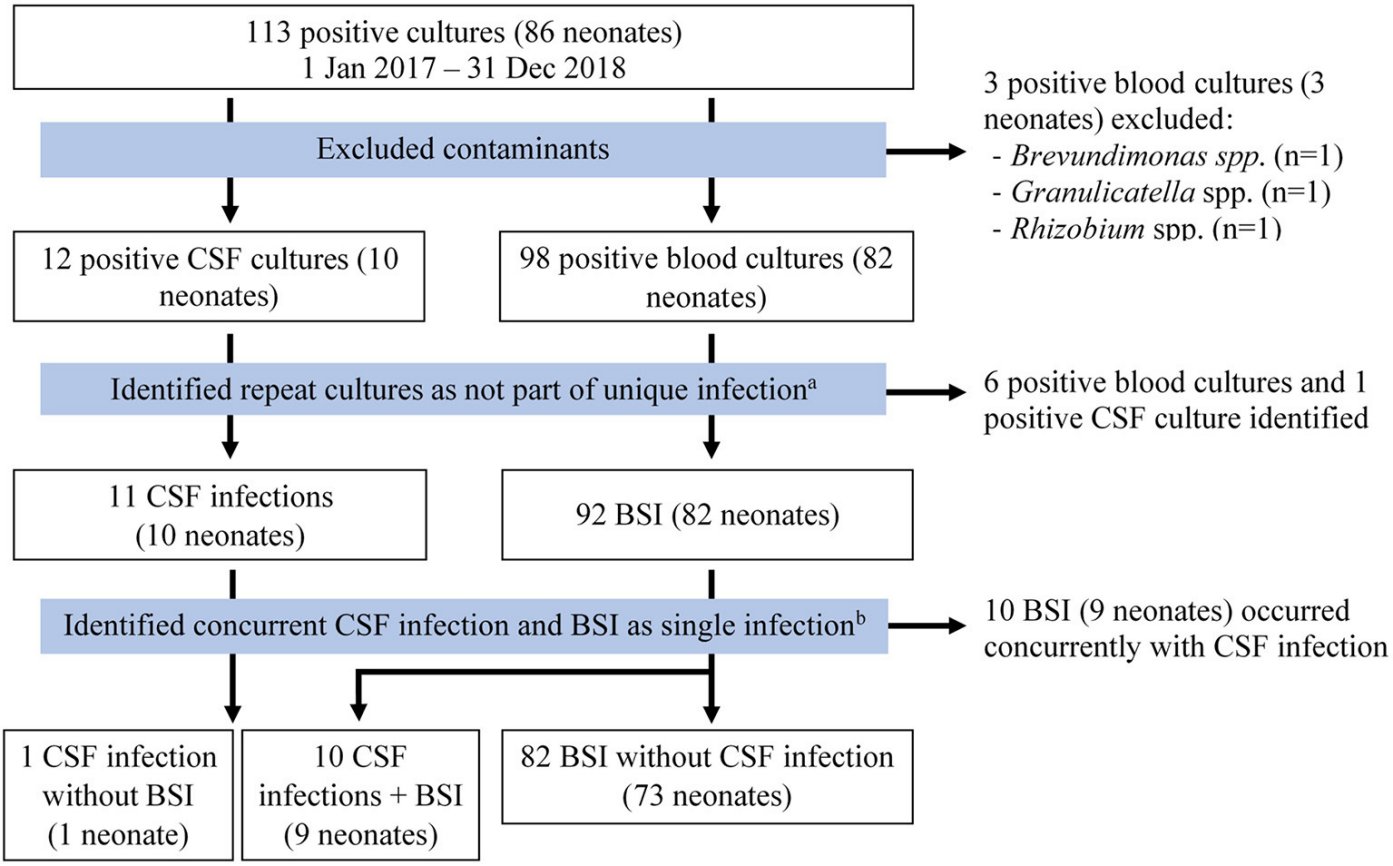
Flow diagram used to identify unique infections. Meningitis and CSF infection were used interchangeably. ^a^Multiple positive cultures from the same neonate were considered part of the same culture-positive infection unless a distinct organism was isolated, or the culture was drawn more than 7 days after the most recent positive culture. ^b^Positive cultures with the same organism from both blood and CSF were considered as part of the same culture-positive infection. BSI, bloodstream infection; CSF, cerebrospinal fluid.

There were 113 positive cultures during the study period, 101 from blood samples and 12 from CSF samples (see Figure 1). Three blood samples were excluded as contaminants [*Brevundimonas* spp. (*n* = 1), *Granulicatella* spp. (*n* = 1), *Rhizobium* spp. (*n* = 1)]. Six blood cultures and one CSF culture from four neonates were positive within 7 days of a prior culture from the same source with the same organism and were considered as part of the same culture-positive infection. Of 11 remaining positive CSF cultures, 10 resulted with the same pathogen as a positive blood culture drawn on the same day and were considered part of the same culture-positive infection. One infant had a positive CSF culture without concurrent positive blood culture. After exclusions, there were 93 culture-positive cases of BSI or meningitis in 83 neonates. Fifty-three percent (49/93) of all infections occurred in males and 84% (78/93) occurred in inborn neonates (Table 1). The median age at birth in completed gestational weeks was 31 [interquartile range (IQR) 29–33] and the median birth weight was 1,340 g (IQR 1,040–1,750). Twenty percent (17/83) of neonates were extremely low birth weight (ELBW, or <1,000 g at birth). Thirty-one percent (26/83) of neonates required invasive ventilation during their hospitalization. Median length of stay was 29 days (IQR 15–41). Fifteen (18%) infants died. Three of four neonates (75%) with documented fungal infections died. Neonates with repeat infections were smaller at birth (median birth weight 1,028 g, IQR 863–1,265) and had prolonged hospitalizations (median length of stay 49 days, IQR 32–62). Of positive cultures from the same neonates, 50% (5/10) were unique infections with the same organism, but minimal changes to susceptibility patterns were noted on AST. The median time between unique infections for these neonates was 14 days (IQR 9–19).

**Table 1:** Clinical and demographic characteristics of neonates with culture-positive bloodstream infections or meningitis.

	**BSI only (*n* = 73)**	**Meningitis (*n* = 10)**	**Total (*n* = 83)**
Maternal age in years, median (IQR)	28 (25–32)	28 (24–30)	28 (24–32)
Female sex, *n* (%)	39 (53)	2 (20)	41 (49)
Gestational age at birth in weeks, median (IQR)	32 (29–33)	30 (29 −31)	31 (29–33)
Premature (<37 weeks), *n* (%)	64 (88)	10 (100)	74 (89)
Birth weight in grams, median (IQR)	1,340 (1,040–1,750)	1,334 (1,200–1,367)	1,340 (1,040–1,750)
ELBW, *n* (%)	16 (22)	1 (10)	17 (20)
IUGR^a^, *n* (%)	18 (25)	1 (10)	19 (23)
Inborn, *n* (%)	62 (85)	8 (80)	70 (84)
Vaginal delivery, *n* (%)	36 (49)	8 (80)	44 (53)
Resuscitation at delivery^b^, *n* (%)	25 (34)	2 (20)	27 (33)
Premature rupture of membranes, *n* (%)	19 (26)	1 (10)	20 (24)
Intrapartum maternal fever (38.5°C or higher), *n* (%)	2 (3)	0 (0)	2 (2)
Antenatal maternal steroid administration, *n* (%)	43 (59)	6 (60)	49 (59)
Meconium-stained amniotic fluid, *n* (%)	1 (1)	0 (0)	1 (1)
Any respiratory support, *n* (%)	49 (67)	4 (40)	53 (64)
Invasive respiratory support, *n* (%)	23 (32)	4 (40)	27 (33)
Any IVH^c^, *n* (%)	13 (18)	2 (20)	15 (18)
Severe IVH (Grade 3 or 4)^c^, *n* (%)	1 (1)	1 (10)	2 (2)
ROP^d^, *n* (%)	10 (14)	0 (0)	10 (12)
NEC^e^, *n* (%)	6 (8)	1 (10)	7 (8)
Hospital stay in days, median (IQR)	25 (14–37)	39 (36–44)	29 (15–41)
Died, *n* (%)	14 (19)	1 (10)	15 (18)

*Clinical and demographic characteristics of neonates with culture proven infection. A total of 83 neonates had culture-proven infections during the study timeframe. Neonates with multiple distinct episodes of infection were included only once*.

a *IUGR defined as less than 10th percentile on gestational age appropriate growth curve*.

b *Resuscitation at delivery defined as need for positive pressure ventilation*.

c *Intraventricular hemorrhage defined according to Papile grading criteria (10)*.

d *Retinopathy of prematurity defined as any stage per International Committee for Classification of Retinopathy of Prematurity criteria (9)*.

e *NEC defined as stage II or greater on modified Bell's criteria (8). BSI, bloodstream infection; ELBW, extremely low birth weight; IQR, interquartile range; IUGR, intrauterine growth restriction; IVH, intraventricular hemorrhage; NEC, necrotizing enterocolitis; ROP, retinopathy of prematurity*.

The majority of infections were late onset (82%, 76/93) with a median age at time of positive culture of 7 days (IQR 4–11) (Table 2). Central lines were present at time of culture in 53% (49/93) of cases. Of infections with a central line present at time of positive culture, the median for total duration of central line use was 6 days (IQR 4–12). The median time to culture positivity (from incubation to growth) was 22 h (IQR 19–25).

**Table 2:** Characteristics of culture-positive infections in early onset and late onset infections.

	**Early onset (*n* = 17)**	**Late onset (*n* = 76)**	**Total (*n* = 93)**
Age at positive culture in days^a^, median (IQR)	2 (1–3)	8 (6–14)	7 (4–11)
Central line at time of culture, *n* (%)	5 (29)	44 (58)	49 (54)
Total duration of central line^b^, median (IQR)	3 (3–4) *n* = 5	8 (4–13.5) *n* = 44	6 (4–12) *n* = 49
Antibiotics at time of culture, *n* (%)	8 (47)	38 (50)	46 (49)
Time to positive culture from incubation in hours, median (IQR)	22 (19–28)	22 (19.5–24.5)	22 (19–25)

*Clinical characteristics related to culture-positive infections in neonates, separated by early onset and late onset infections. Early onset infections were defined as occurring within 72 h of birth and late onset infections were defined as occurring after 72 h of life*.

a *Date of birth reported as day of life 1*.

b *Total duration of central line reflects total duration of central line presence, not duration at time of culture. IQR, interquartile range*.

Gram-negative infections were predominant (85%, 79/93) (Table 3). The most commonly isolated Gram-negative pathogens were *Klebsiella pneumoniae* (52% of all infections, 48/93), *Acinetobacter* spp. (15%, 14/93), and *Escherichia coli* (9%, 8/93). *Enterococcus faecalis* accounted for 60% of Gram-positive infections (6/10) and 6% of all infections (6/93). There were four fungal infections, all caused by *Candida albicans*.

**Table 3:** Distribution of pathogens in early onset and late onset culture-positive infections.

	**Early onset (*n* = 17)**	**Late onset (*n* = 76)**	**Total (*n* = 93)**
**Gram-negative organisms**, ***n*** **(%)**	14 (82)	65 (86)	79 (85)
*Klebsiella pneumonia*	7 (41)	41 (54)	48 (52)
*Acinetobacter* spp.	2 (12)	12 (16)	14 (15)
*Escherichia coli*	2 (12)	6 (8)	8 (9)
*Elizabethkingia* spp.	0 (0)	3 (4)	3 (3)
*Serratia marcescens*	1 (6)	2 (3)	3 (3)
*Enterobacter cloacae*	0 (0)	1 (1)	1 (1)
*Bulkholderia cepacia*	1 (6)	0 (0)	1 (1)
*Moraxella lacunata*	1 (6)	0 (0)	1 (1)
**Gram-positive organisms**, ***n*** **(%)**	2 (12)	8 (11)	10 (11)
*Enterococcus faecalis*	1 (6)	5 (7)	6 (6)
Coagulase-negative *Staphylococcus* spp.	1 (6)	2 (2)	3 (3)
*Staphylococcus aureus*	0 (0)	1 (1)	1 (1)
**Fungal organisms**, ***n*** **(%)**	1 (6)	3 (4)	4 (4)
*Candida albicans*	1 (6)	3 (4)	4 (4)

*Pathogen distribution for all culture-positive infections, identified by early or late onset infections. Early onset infections were defined as occurring within 72 h of birth and late onset infections were defined as occurring after 72 h of life*.

Among the 10 neonates with meningitis, one neonate had two separate CSF infections (18 days apart). Neonates with meningitis were predominantly male (80%, 8/10); all 10 (100%) were premature. Of 11 cases of meningitis, 82% were late onset; the median day of life for a positive CSF culture was 10 days (IQR 4–20). Infants with meningitis had prolonged hospitalizations compared to the entire cohort (39 days, IQR 36–44). Only one (10%) neonate with meningitis died. Pathogens isolated from CSF cultures included *E. faecalis* (*n* = 3, 27%), *K. pneumoniae* (*n* = 3, 27%), *Acinetobacter* spp. (*n* = 2, 18%), *Serratia marcescens* (*n* = 1, 9%)*, Elizabethkingia* spp. (*n* = 1, 9%), and *Moraxella lacunata* (*n* = 1, 9%). Only one infant, who developed *S. marcescens* meningitis on day of life three, did not have concurrent BSI with the same organism.

Among pathogens causing BSI or meningitis, AMR was common. For all Gram-negative isolates, resistance to commonly used empiric antibiotic classes such as aminoglycosides and third-generation cephalosporins was reported in 74% (57 of 77 isolates tested) and 95% (73/77), respectively (Table 4). Gentamicin was less effective than amikacin; 67% (58/87) of samples were resistant to gentamicin compared to 26% (17/66) that were resistant to amikacin. Carbapenem resistance was 56% (42/75); resistance to fluoroquinolones was 91% (70/77). Resistance to colistin was reported in six of 44 isolates tested (14%), all six of which were *K. pneumoniae*, corresponding to 30% colistin resistance for the most commonly isolated pathogen in our cohort.

**Table 4:** Antimicrobial susceptibility testing for culture-positive Gram-negative infections.

	**Aminoglycoside^a^**	**Fluoroquinolone^b^**	**3rd/4th generation**	**Carbapenem^d^**	**Colistin**
	**resistance**	**resistance**	**cephalosporin^c^**	**resistance**	**resistance**
	**(*n* = 78)**	**(*n* = 77)**	**resistance (*n* = 77)**	**(*n* = 75)**	**(*n* = 41)**
Gram-negative organisms	57/78 (73)	70/77 (91)	72/77 (94)	42/75 (56)	6/41 (14)
*n* resistant/isolates tested (%)					
*Klebsiella pneumoniae*	32/47 (68)	43/46 (93)	45/46 (98)	26/46 (57)	6/20 (30)
*Acinetobacter* spp.	13/14 (93)	13/14 (93)	13/14 (93)	12/13 (92)	0/14 (0)
*Escherichia coli*	8/8 (100)	8/8 (100)	8/8 (100)	0/8 (0)	0/5 (0)
*Elizabethkingia* spp.	3/3 (100)	2/3 (67)	3/3 (100)	3/3 (100)	–
*Serratia marcescens*	1/3 (33)	3/3 (100)	2/3 (67)	1/2 (50)	–
*Enterobacter cloacae*	0/1 (0)	0/1 (0)	0/1 (0)	0/1 (0)	0/1 (0)
*Bulkholderia cepacia*	0/1 (0)	1/1 (100)	1/1 (100)	0/1 (0)	–
*Moraxella lacunata*	0/1 (0)	0/1 (0)	0/1 (0)	0/1 (0)	0/1 (0)

*Antimicrobial susceptibility testing reported by pathogen. Resistance defined as intermediate susceptibility or resistance. Resistance to a class of antibiotics described as resistance to one or more antibiotics tested in that class. For infants with identical pathogens identified from positive CSF and blood cultures on the same day, AST testing reported only once. Percentages calculated with number of resistant isolates as numerator and number of isolates tested as denominator. Dashes indicate testing was not done on these isolates*.

a *Aminoglycoside antibiotics tested were amikacin and gentamicin*.

b *Fluoroquinolone antibiotics tested were ciprofloxacin and levofloxacin*.

c *3rd and 4th generation cephalosporin antibiotics tested were cefoperazone/sulbactam, ceftazidime, ceftriaxone, and cefepime*.

d *Carbapenem antibiotics tested were doripenem, meropenem, and imipenem*.

Resistance to carbapenems was seen in 46% (6/13) of early onset infections and 58% (36/62) of late onset infections. Although rates of resistance to aminoglycosides were similar between early onset and late onset infections (67%, 11/16 vs. 74%, 53/72, respectively), this similarity was less notable for amikacin. Resistance to amikacin was seen in 8% (1/12) early onset infections and 29% (16/55) late onset infections. Rates of resistance to 3rd/4th generation cephalosporins (86%, 12/14 vs. 95%, 61/64), fluoroquinolones (93%, 13/14 vs. 91%, 58/64), and colistin (14%, 1/7 vs. 14%, 5/37) were similar in early onset infections and late onset infections.

Although Gram-negative infections were predominant, there were 10 Gram-positive infections and four blood cultures positive for *C. albicans*. All *E. faecalis* samples were susceptible to vancomycin and linezolid, but resistant to clindamycin. The four fungal isolates were susceptible to all antifungal therapies tested (amphotericin-B, caspofungin, fluconazole, flucytosine, and micafungin).

Among clinical characteristics of neonates, only ELBW [odds ratio (OR) 6.0, confidence interval (CI) 1.9–18.7, *p* = 0.002], invasive respiratory support (OR 7.7, CI 2.4–24.9, *p* = 0.001), and antibiotic use at time of culture (OR 4.2, CI 1.3–14.2, *p* = 0.019) were associated with an increased risk of mortality (Table 5). There were no deaths among neonates with colistin-resistant infections. Carbapenem resistance was not associated with mortality among all neonates. A sub-analysis of only preterm neonates (*n* = 74) was done. Clinical characteristics and AST testing of the 84 infections were similar compared to the entire cohort. In preterm infants, the presence of a central line was also associated with mortality (OR 5.4, CI 1.1–25.8, *p* = 0.037).

**Table 5:** Association of clinical characteristics and carbapenem resistance with mortality.

	**Mortality**		
	**OR**	**95% CI**	***p*** **-value**
Prematurity	0.23	0.05–0.96	0.05
Male sex	0.57	0.20–1.64	0.30
Antenatal steroids	0.61	0.21–1.76	0.36
Intrapartum maternal fever	^*^	^*^	^*^
Meconium-stained fluids at birth	^*^	^*^	^*^
ELBW	**6.0**	**1.93–18.66**	**0.002**
Vaginal delivery	1.36	0.47–3.93	0.58
Inborn	0.65	0.13–3.17	0.59
Invasive respiratory support	**7.73**	**2.40–24.91**	**0.001**
PROM	0.81	0.24–2.75	0.73
Resuscitation at birth	1.9	0.66–5.60	0.23
Central line at time of culture	2.6	0.81–7.88	0.11
IUGR	1.45	0.45–4.68	0.54
Antibiotics at time of culture	**4.23**	**1.26–14.19**	**0.02**
Gestational diabetes	0.47	0.05–3.94	0.48
Gestational hypertension	0.99	0.33–2.97	0.98
Carbapenem resistance	1.52	0.46–5.09	0.49
Doripenem resistance	0.70	0.15–3.2	0.64
Meropenem resistance	0.94	0.29–2.99	0.91
Imipenem resistance	1.47	0.44–4.92	0.53

*Univariate analysis for baseline clinical characteristics, carbapenem resistance, and mortality*.

* *Odds ratios unable to be calculated in these cases given small sample sizes and lack of neonates in all groups. CI, confidence interval; ELBW, extremely low birth weight; IUGR, intrauterine growth restriction; OR, odds ratio; PROM, premature rupture of membranes. Bold values are used to denote associations with p-values < 0.05*.

## Discussion

Gram-negative infections predominate in our study, with 52% of all infections caused by *K. pneumoniae* and 14% caused by *Acinetobacter* spp. This epidemiologic profile mirrors prior hospital data reported in India where Gram-negative infections (especially *K. pneumoniae* and *Acinetobacter* spp.) account for 60–70% of all culture proven infections in neonates admitted to NICUs (3, 6). The high rate of *K. pneumoniae* infections in our study aligns with prior data from our institution (41% in 2006–2008) (5).

This study includes detailed AST data from a large number of isolates. Unfortunately, we report a high incidence of AMR to commonly used and broad-spectrum antibiotics. The majority of isolates were resistant to third-generation cephalosporins, aminoglycosides, and carbapenems. Resistance to carbapenems varied widely depending on the organism isolated. *Acinetobacter* spp., a major health-care associated pathogen in India capable of acquiring resistance, was nearly universally resistant to carbapenems in our study (11). There were similar levels of resistance to all drugs within most antibiotic classes, with the exception of aminoglycosides; isolates showed more susceptibility to amikacin, compared to gentamicin. This discrepancy exists in other areas of India as well as in other LMIC countries (12–14). The high rate of resistance to gentamicin likely is influenced by widespread use driven by its inclusion as a first-line antibiotic for neonatal sepsis in the WHO's guidelines (15). Institutions such as our own, which are routinely using amikacin as the preferred aminoglycoside instead of gentamicin, may find themselves limited in the future as AMR to efficacious antibiotics increases with increased use. This highlights the need not only to select appropriate antibiotics, but to prioritize antimicrobial stewardship activities to reduce overall antibiotic use.

Rates of resistance to antibiotic classes or single agents (excepting amikacin) were similar among early onset infections and late onset infections. A higher rate of resistance to amikacin in late onset infections may suggest that nosocomial spread contributes to rising rates of AMR or that early exposure to amikacin confers resistance in later infections. Transmission of AMR infections may be driven by multiple reservoirs of transmission, including maternal colonization with community-acquired pathogens followed by vertical transmission or rapid colonization after birth and subsequent infection. Better understanding of the differences in epidemiology between early and late onset neonatal infections is required to direct infection prevention practices and antimicrobial stewardship initiatives. At KEMHRC, multidisciplinary infection prevention and control (IPC) efforts are supported by dedicated nursing staff and include a focus on staff education, hand-hygiene, environmental surveillance, and outbreak investigations.

Notably, a central line was present more often and used for longer durations in late-onset BSIs compared to early-onset BSIs. Our study was not designed to assess whether the reported BSIs were central-line associated blood stream infections, but highlights an opportunity for tailoring IPC practices. Central lines are a known risk factor for BSI and that risk changes over the lifetime of the central line. At KEMHRC, umbilical catheters are not used beyond 7 days of life, and infants with expected prolonged need for central access preferentially undergo peripherally inserted central catheter (PICC) placement. IPC practices should account for the different risks associated with different phases of central line use (insertion, compared to maintenance). Some universal practices which apply to all phases (and all patients) include effective hand hygiene before and after every patient encounter and aseptic technique when inserting or accessing a central line. Daily discussion of line necessity and prompt removal of central lines when appropriate is an integral component of central line associated BSI prevention (16).

Prior to the rise in difficult-to-treat, MDR infections, colistin, a polymyxin antibiotic with activity against Gram-negative infections, was routinely avoided given paucity of data in neonates and concerns for difficult pharmacokinetics, nephrotoxicity, and neurotoxicity (17, 18). In India, clinicians over the past 10 years are turning more frequently to colistin as part of routine practice (19). Worryingly, our study showed higher rates of colistin resistance (14% overall, 30% in *K. pneumoniae* isolates) than previously reported in the literature on neonates in India (0–10%) (3, 20–25). Polymyxins are classified as a “reserve” antibiotic class by the WHO (26). Unfortunately, there is mounting evidence that increased colistin use drives colistin resistance, especially in *K. pneumoniae*. Epidemiologic data from a 2017 report by the European Center for Disease Prevention and Control on polymyxin use in 30 European countries reported an association between total consumption of polymyxin antibiotics and resistance in *K. pneumoniae* (27). In studies of hospitalized adult patients, use of colistin was a risk factor for infection with colistin resistant *K. pneumoniae* and there are reports of patients developing colistin resistance during colistin therapy (28–30). More concerning, once resistance in *K. pneumoniae* isolates develops, resistant infections have been shown to spread rapidly *via* clonal spread and biofilm formation, including in neonates (31–34).

Limiting colistin use and effective IPC programs to prevent spread of resistance are necessary to prevent further increases in resistance. Even when colistin use is appropriately limited to MDR infections, how it is used can potentially create conditions that lead to increased resistance. While colistin monotherapy is traditionally used, some question whether a combination regimen that includes colistin with possible synergistic effects would be more effective or even protective against colistin resistance (35, 36). Adequate dosing in MDR infections remains key as sub-optimal dosing or prolonged therapy has been shown to increase the risk for acquired resistance, including in neonates (30). Addressing the rising trend in colistin resistance will require comprehensive approaches including stewardship programs, IPC programs, and improved education about colistin use. Important to all of these initiatives will be improved surveillance for colistin resistance. Colistin resistance was not universally tested for in our study. However, given the increased risk of emerging resistance, clinicians and microbiologists should consider routinely testing for colistin on AST.

Limitations for this study include its retrospective nature, focus on a single center in western India, and lack of confirmatory cultures for potential contaminant pathogens. In our study, *Brevundimonas* spp., *Granulicatella* spp., and *Rhizobium* spp. were excluded as likely contaminants. As prior case reports have reported true infections in neonates with these pathogens, we may have excluded potential real infections (37–39). Coagulase negative staphylococcus (CoNS) isolates were included as infections despite lack of confirmatory culture given the known role of CoNS as a neonatal pathogen, especially in late onset neonatal infections (40).

This study adds awareness to the growing concern about rising AMR in neonatal infections in India. The lack of narrow empiric regimens with adequate coverage for neonatal sepsis leads to clinicians using broader spectrum agents as empiric therapy, which in turn, leads to increasing resistance. As use of last-resort antibiotics increases, clinicians must remain vigilant in prescribing practices. Standardization of empiric antibiotic therapy, avoiding overlapping therapy, and timely reevaluation of the antibiotic regimen (including narrowing coverage when appropriate and stopping antibiotics in neonates with negative cultures) are practical areas of focus for neonatal practitioners. Strengthening IPC practices and antimicrobial stewardship programs will be paramount in reducing the burden of AMR neonatal sepsis in India and other LMIC.

## Data Availability Statement

The raw data supporting the conclusions of this article will be made available by the authors, without undue reservation.

## Ethics Statement

The studies involving human participants were reviewed and approved by King Edward Memorial Hospital Research Center Ethics Committee. Written informed consent from the participants' legal guardian/next of kin was not required to participate in this study in accordance with the national legislation and the institutional requirements.

## Author Contributions

MS: conceptualization, methodology, data collection, and data management. SM: literature review, data analysis, and writing—original draft, review, and editing. SK: conceptualization. TP: conceptualization and data collection. UV: project administration. SS: data collection. JJ: conceptualization, data analysis, and writing—review and editing. All authors have approved the submitted version.

## Funding

JJ was supported by the National Institutes of Health (K23HD100594).

## Conflict of Interest

The authors declare that the research was conducted in the absence of any commercial or financial relationships that could be construed as a potential conflict of interest.

## Publisher's Note

All claims expressed in this article are solely those of the authors and do not necessarily represent those of their affiliated organizations, or those of the publisher, the editors and the reviewers. Any product that may be evaluated in this article, or claim that may be made by its manufacturer, is not guaranteed or endorsed by the publisher.
