# Supplementary material for: Emerging Antibiotic Resistance Patterns in a Neonatal Intensive Care Unit in Pune, India: A 2-Year Retrospective Study
Source: Front Pediatr. 2022 Jun 10;10:864115. doi: 10.3389/fped.2022.864115 (PMC9226713; doi:10.3389/fped.2022.864115)
Supplement: Supplementary file 1 [file Table_1.DOCX]

Supplemental Table 1. Antimicrobial resistance for antibiotics tested for all Gram-negative pathogens isolated

|  |  | Amikacin | Gentamicin | Minocycline | Tigecycline | Piperacillin-Tazobactam | Cefoperazone-Sulbactam | Ceftazidime | Cefepime | Aztreonam | Ciprofloxacin | Levofloxacin | Doripenem | Imipenem | Meropenem | Colistin | Co-trimoxazole |
| --- | --- | --- | --- | --- | --- | --- | --- | --- | --- | --- | --- | --- | --- | --- | --- | --- | --- |
| Gram-negative | |  |  |  |  |  |  |  |  |  |  |  |  |  |  |  |  |
|  | *Acinetobacter* spp. | 3/4 | 13/14 | 8/14 | 10/14 | 13/14 | 9/14 | 11/12 | 13/14 | 13/14 | 13/14 | 7/7 | 3/4 | 12/13 | 12/13 | 0/14 | 4/14 |
|  | *Bulkholderia cepacia* | 0/1 | 0/1 | 0/1 | - | 0/1 | 0/1 | 1/1 | 1/1 | 1/1 | 0/1 | 1/1 | 0/1 | 0/1 | 0/1 | - | 0/1 |
|  | *Escherichia coli* | 0/8 | 8/8 | 0/3 | 0/8 | 0/8 | 0/8 | 8/8 | 8/8 | 8/8 | 8/8 | 3/3 | 0/8 | 0/8 | 0/8 | 0/5 | 5/8 |
|  | *Elizabethkingia* spp. | 3/3 | 3/3 | 0/3 | 2/3 | 3/3 | 3/3 | 3/3 | 3/3 | 3/3 | 2/3 | 0/3 |  | 3/3 | 3/3 | - | 3/3 |
|  | *Enterobacter cloacae* | 0/1 | 0/1 | 0/1 | 0/1 | 0/1 | 0/1 | 0/1 | 0/1 | 0/1 | 0/1 | - | 0/1 | 0/1 | 0/1 | 0/1 | 0/1 |
|  | *Klebsiella pneumoniae* | 11/46 | 26/47 | 40/46 | 19/29 | 39/46 | 34/46 | 45/46 | 40/46 | 45/46 | 41/46 | 24/26 | 24/45 | 26/46 | 23/46 | 6/20 | 16/47 |
|  | *Moraxella lacunata* | 0/1 | 0/1 | 0/1 | 0/1 | 0/1 | 0/1 | 0/1 | 0/1 | 0/1 | 0/1 | 0/1 | 0/1 | 0/1 | 0/1 | 0/1 | 0/1 |
|  | *Serratia marcescens* | 0/3 | 1/3 | 2/3 | 0/2 | - | 1/3 | 2/3 | 2/3 | 2/3 | 1/3 | 2/2 | 1/2 | 1/1 | 0/1 | - | 0/2 |

Table notes. Antimicrobial resistance to all antibiotics tested by Gram-negative pathogen isolated. Resistance defined as ‘resistant’ or ‘intermediate susceptibility’ on antimicrobial susceptibility testing. Numerator is number of resistant isolates, denominator is number of tested isolates. Not every sample was tested for all antibiotics.
