# Supplementary material for: Emerging Antibiotic Resistance Patterns in a Neonatal Intensive Care Unit in Pune, India: A 2-Year Retrospective Study
Source: Front Pediatr. 2022 Jun 10;10:864115. doi: 10.3389/fped.2022.864115 (PMC9226713; doi:10.3389/fped.2022.864115)
Supplement: Supplementary file 2 [file Table_2.DOCX]

Supplemental Table 2. Antimicrobial resistance for antibiotics tested for all Gram-positive pathogens isolated

|  |  | Gentamicin | Tetracycline | Penicillin-G | Oxacillin | Methicillin | Amoxicillin-clavulanate | Cefoperazone-Sulbactam | Ceftriaxone | Cefepime | Cefuroxime | Ciprofloxacin | Levofloxacin | Vancomycin | Linezolid | Daptomycin | Clindamycin | Teicoplanin | Colistin | Co-trimoxazole | Erythromycin | Rifampin |
| --- | --- | --- | --- | --- | --- | --- | --- | --- | --- | --- | --- | --- | --- | --- | --- | --- | --- | --- | --- | --- | --- | --- |
| Gram-positive | |  |  |  |  |  |  |  |  |  |  |  |  |  |  |  |  |  |  |  |  |  |
|  | *Enterococcus faecalis* | 6/6 | 1/3 | 5/5 | - | - | - | - | - | - | - | - | - | 0/6 | 0/6 | 0/1 | 5/5 | 0/6 | 0/3 | - | 6/6 | - |
|  | *Staphylococcus aureus* | 1/1 | 0/1 | - | - | - | 1/1 | 1/1 | 1/1 | 1/1 | 1/1 | 1/1 | 1/1 | 0/1 | 0/1 | 0/1 | - | 0/1 | - | 0/1 | - | - |
|  | *Staphylococcus epidermidis* | 0/2 | 2/2 | 2/2 | 2/2 | 0/2 | - | - | - | - | - | - | - | 0/2 | 0/2 | 2/2 | 2/2 | 0/2 | - | - | 2/2 | 2/2 |
|  | *Staphylococcus xylosus* | 0/1 | 1/1 | 1/1 | 2/2 | 0/1 | - | - | - | - | - | - | - | 0/1 | 0/1 | 1/1 | 1/1 | 0/1 | - | - | 1/1 | 1/1 |

Table notes. Antimicrobial resistance to all antibiotics tested by pathogen isolated. Resistance defined as ‘resistant’ or ‘intermediate susceptibility’ on antimicrobial susceptibility testing. Numerator is number of resistant isolates, denominator is number of tested isolates. Not every sample was tested for all antibiotics.
